# Loss of miR-200b promotes invasion via activating the Kindlin-2/integrin β1/AKT pathway in esophageal squamous cell carcinoma: An E-cadherin-independent mechanism

**DOI:** 10.18632/oncotarget.5027

**Published:** 2015-08-20

**Authors:** Hai-Feng Zhang, Abdulraheem Alshareef, Chengsheng Wu, Shang Li, Ji-Wei Jiao, Hui-Hui Cao, Raymond Lai, Li-Yan Xu, En-Min Li

**Affiliations:** ^1^ The Key Laboratory of Molecular Biology for High Cancer Incidence Coastal Chaoshan Area, Shantou University Medical College, Shantou, Guangdong, China; ^2^ Institute of Oncologic Pathology, Shantou University Medical College, Shantou, Guangdong, China; ^3^ Department of Laboratory Medicine and Pathology, University of Alberta, Edmonton, Alberta, Canada; ^4^ College of Bioinformatics Science and Technology, Harbin Medical University, Harbin, Heilongjiang, China

**Keywords:** miR-200, E-cadherin, invasion, prognosis, esophageal squamous cell carcinoma

## Abstract

Our previous studies have shown that loss of miR-200b enhances the invasiveness of esophageal squamous cell carcinoma (ESCC) cells. However, whether the miR-200-ZEB1/2-E-cadherin regulatory cascade, a master regulator of epithelial-to-mesenchymal transition (EMT), is involved in the regulation of ESCC invasion remains elusive. Here, we show that miR-200b represses ESCC cell invasion *in vivo* without altering the expression of E-cadherin and vimentin, two surrogate markers of EMT. However, an inverse correlation was observed between the expression levels of miR-200b and ZEB1/2 in both ESCC cell lines (*n* = 7, *P* < 0.05) and ESCC tumor samples (*n* = 88, *P* < 0.05). Methylation of *E-cadherin* gene was found to block the regulation of E-cadherin by the miR-200b-ZEB1/2 axis, indicating that an E-cadherin-independent mechanism can mediate the biological function of miR-200b in ESCC. We revealed that miR-200b suppresses the integrin β1-AKT pathway via targeting Kindlin-2 to mitigate ESCC cell invasiveness. In two independent cohorts of ESCC samples (*n* = 20 and *n* = 53, respectively), Kindlin-2 expression positively correlated with the activation status of both the integrin signaling pathway and the PI3K-AKT signaling pathway (both *P* < 0.01). These data highlight that suppression of the Kindlin-2-integrin β1-AKT regulatory axis is an alternative mechanism underlying the tumor suppressor function of miR-200b in ESCC.

## INTRODUCTION

Esophageal cancer, one of the deadliest malignancies, inflicts an estimate of 480,000 new patients worldwide annually, and it represents the sixth leading cause of cancer-related deaths [1–2]. Virtually all cases of esophageal cancer in China are esophageal squamous cell carcinoma (ESCC) [1–2]. The overall 5-year survival rate for ESCC patients is only 14% [3–5], mainly attributed to the fact that > 50% of patients have developed radiographically visible metastases at time of initial diagnosis [3–6]. Thus, understanding the molecular mechanisms underlying the invasiveness and metastasis of ESCC carries great importance.

The miR-200 family comprises 5 members that are encoded by two clusters of genes, *i.e*. the miR-200b/200a/429 cluster on Chr.1p36 and the miR-200c-141 cluster on Chr.12p13. Deregulation of miR-200 has been shown to suppress invasion/metastasis in various cancer types, mainly by preventing epithelial-to-mesenchymal transition (EMT) [7–12]. Specifically, miR-200 prevents EMT via directly targeting and inhibiting the expression of multiple transcriptional repressors of E-cadherin, including ZEB1, ZEB2 and SLUG [7, 13, 14]. Notably, the self-strengthening feedback loop formed between miR-200 and ZEB1/2 has been demonstrated as a master regulator of EMT [7]. miR-200 can also inhibit invasion/metastasis via attenuating the activity of key regulators of actin cytoskeketon remodeling, including RhoA and CDC42 [11], and repressing a network of cytoskeleton regulators, including WASF3, Moesin, WIPF1 and Cofilin2 [8–12].

Our previous studies have reported that miR-200b suppresses the invasiveness of ESCC *in vitro* via repressing the cytoskeletal and the adhesive machinery, and Kindlin-2 was identified as a direct target and functional mediator of miR-200b [11]. Nevertheless, the *in vivo* function of miR-200b in ESCC invasion is still unclear. Moreover, the pathobiological functions of the miR-200b-ZEB1/2 feedback loop and its regulation on EMT remain elusive in ESCC. In this study, we revealed that the miR-200b-ZEB1/2 axis contributes to the pathobiology of ESCC via an EMT-independent mechanism, whereas suppression of the Kindlin-2-integrin β1-AKT regulatory axis is an alternative mechanism underlying the tumor suppressor function of miR-200b in ESCC.

## RESULTS

### miR-200b suppresses ESCC tumor invasion *in vivo*

**Figure 1:**
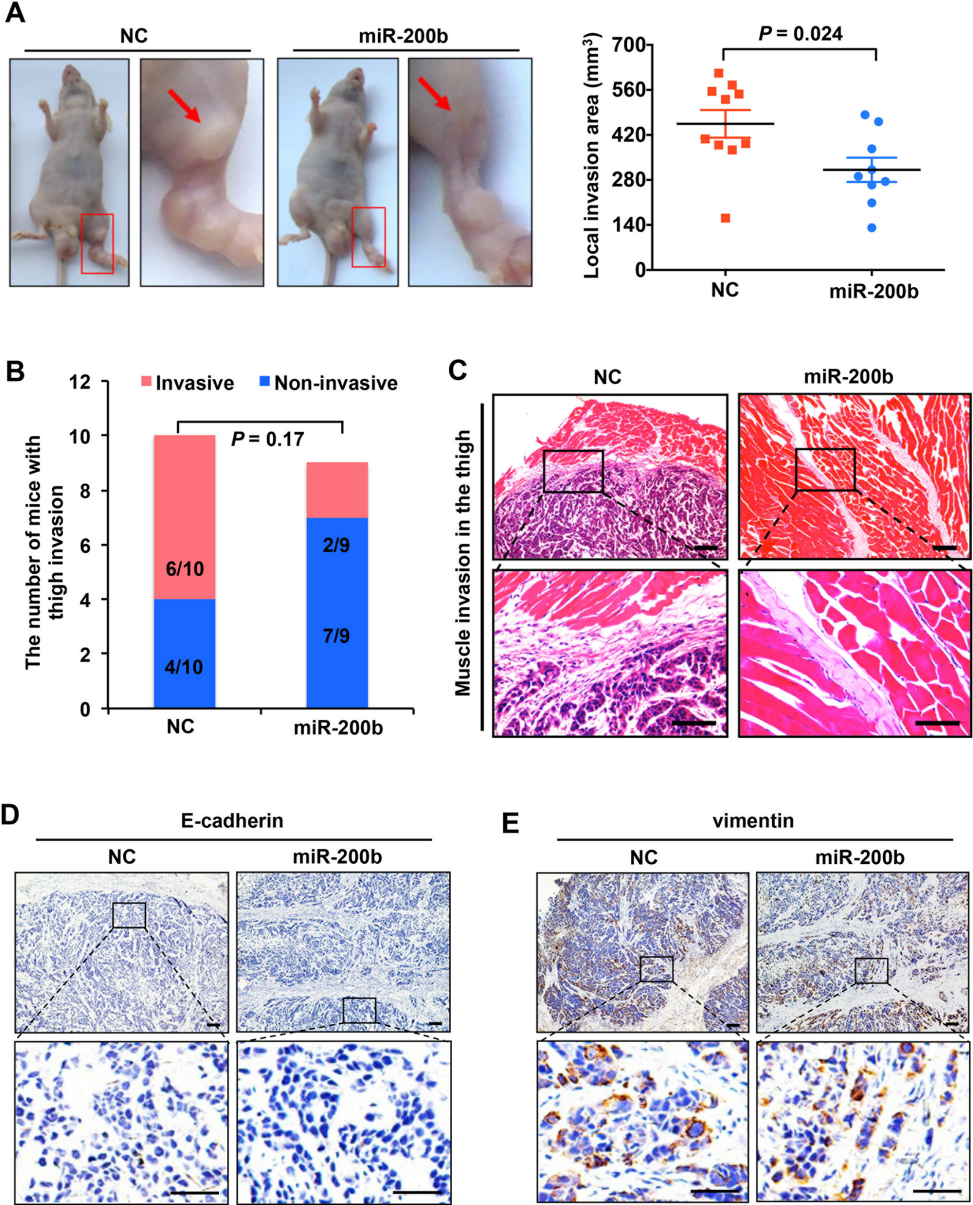
miR-200b suppresses ESCC tumor invasion *in vivo* **A.** Left panel: representative mice showing the impact of miR-200b on ESCC tumor invasion *in vivo*. Note that the invasion sites on the thigh were magnified, and the arrows indicate the invaded tumor nodules. Right panel: the local invasion areas of tumors formed by EC109-NC and EC109–200b cells were compared. Data are presented as mean ± SD. Student's *t* test was used in the statistical analysis. **B.** The proportions of mice that developed invasive tumors in the thigh region were compared. *n* = 10 for NC transfected group, and *n* = 9 for miR-200b transfected group. **C.** Representative H&E staining showing the invaded tumor nodules within the muscle of the thigh area. Upper panel scale bars: 100 μm; lower panel scale bars: 50 μm. **D–E.** Immunostaining of E-cadherin and vimentin in tumor xenografts dissected from the mice. Upper panel scale bars: 100 μm; lower panel scale bars: 50 μm.

Our previous study has shown that miR-200b impairs ESCC cell migration and invasion *in vitro* [11]. In this study, we asked if miR-200b also affects the tumor invasiveness of ESCC *in vivo* using a previously described mouse model [15]. EC109, a miR-200b-low ESCC cell line [11], was transfected with either a miR-200b mimic or negative control RNA, and these cells were injected into the left footpads of mice. As shown in Figure 1A, enforced expression of miR-200b significantly reduced the local invasion area compared with the negative control (*P* = 0.024). Notably, invasive tumor nodules were found in the unilateral thigh region in a higher proportion of mice in the negative control group (6/10) compared to the miR-200b-transfected group (2/9) (Figure 1A–1C), even though the difference does not reach statistical significance (*P* = 0.17). As shown in Figure 1D–1E, despite the inhibitory effect of miR-200b in the tumor invasion of ESCC, the expression of neither E-cadherin nor vimentin was appreciably altered by the miR-200b mimic in the xenografts.

### The miR-200b-ZEB1/2 axis contributes to the pathobiology of ESCC

**Figure 2:**
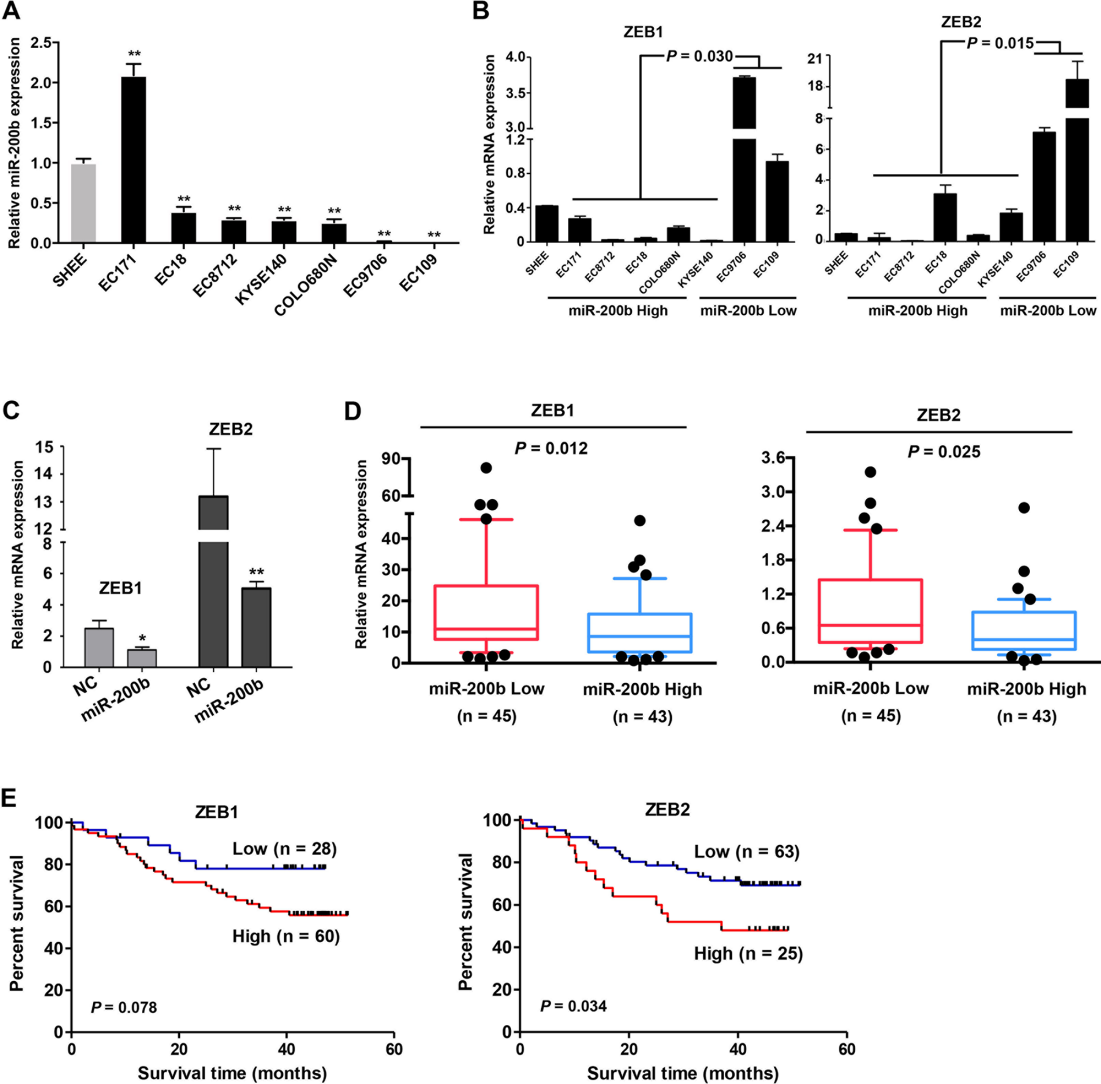
The miR-200b-ZEB1/2 axis in ESCC cell lines and patient tumors **A–B.** The expression of miR-200b and ZEB1/2 were determined using real-time PCR in a panel of cell lines. SHEE, an immortalized cell line was used as a normal control. Data are presented as mean ± SD. **P < 0.01, Student's *t* test. **C.** In EC109 cells, the influence of the enforced expression of miR-200b on the expression of ZEB1/2 mRNA was determined using real-time PCR. **D.** The expression of ZEB1 and ZEB2 mRNA were compared between ESCC tumors expressing low or high levels of miR-200b (Median miR-200b expression level was used in the stratification). Mann Whitney *U* test was used in the statistical analysis. **E.** The association of ZEB1 and ZEB2 expression with the overall survival of ESCC patients was analyzed using Kaplan-Meier survival analysis, and log-rank test was used in the statistical analysis.

We then asked whether the tumor suppressor effects of miR-200b were mediated via the miR-200b-ZEB1/ 2-E-cadherin axis. Firstly, the pathological significance of the miR-200b-ZEB1/2 axis in ESCC was assessed. As shown in Figure 2A–2B, the expression of miR-200b was substantially decreased in 6/7 ESCC cell lines as compared to an immortalized esophageal cell line (*P* < 0.01), and a low expression of miR-200b was associated with a high expression of ZEB1/2 mRNA in this panel of ESCC cell lines (*P* < 0.05). Enforced expression of miR-200b in EC109, a miR-200b-low cell line, dramatically decreased the expression of ZEB1/2 in these cells (Figure 2C). Furthermore, in a cohort of 88 ESCC tumor samples with which the expression status of miR-200b had been examined in our previous study [11], we found a significant inverse correlation between the expression levels of ZEB1/2 and that of miR-200b (*P* < 0.05) (Figure 2D). Correlating with our previous finding that a low expression of miR-200b was associated with a poor prognosis in ESCC patients [11], we found that a high expression of ZEB2 significantly correlated with a shorter overall survival (*P* = 0.034), although the correlation between ZEB1 and survival just fell short of statistical significance (*P* = 0.078) (Figure 2E). These data suggest that deregulation of the miR-200b-ZEB1/2 axis is involved in the pathobiology of ESCC.

### E-cadherin is not a critical mediator of the miR-200b-ZEB1/2 axis in ESCC

**Figure 3:**
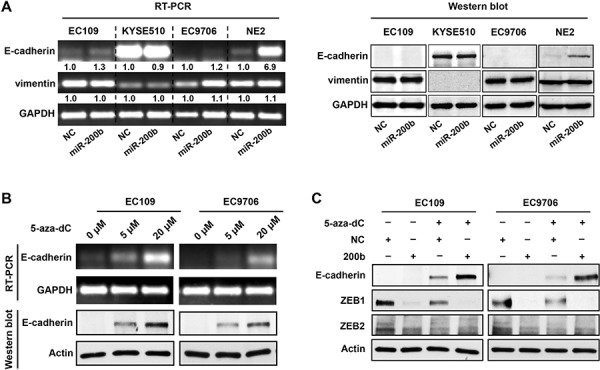
DNA methylation of *E-cadherin* gene blocks the control of E-cadherin expression by the miR-200b-ZEB1/2 axis **A.** RT-PCR and Western blot assays were performed to determine the impact of miR-200b mimic transfection on the expression of E-cadherin and vimentin in ESCC cell lines (EC109, KYSE510 and EC9706) and an immortalized esophageal cell line NE2. **B.** RT-PCR and Western blot assays were performed to determine the impact of 5-aza-dC treatment (72 h) on the expression of E-cadherin and vimentin in EC109 and EC9706 cells. GAPDH and Actin were used as loading controls for RT-PCR and Western blot, respectively. **C.** Western blot was performed to determine the impact of 5-aza-dC treatment and/or miR-200b mimic transfection on the expression of E-cadherin. Actin was used as a loading control.

We then determined whether E-cadherin, a key downstream mediator of the miR-200b-ZEB1/2 axis, mediates the biological function of miR-200b in ESCC. Although transfection of the miR-200b mimic induced dramatic morphological changes in EC109 and EC9706 cells (Supplementary Figure 1A–1B), it did not increase the protein expression of E-cadherin, and only slightly increased its mRNA (Figure 3A). Moreover, using immunohistochemistry applied to 37 cases of ESCC tumor samples, we found no significant correlation between the E-cadherin and miR-200b or ZEB1/2 (Supplementary Figure 2A–2B). These results were further confirmed by Western blot analysis (Supplementary Figure 2C–2D). This lack of correlation between E-cadherin and miR-200b or ZEB1/2 is probably due to the fact that E-cadherin has been reported to be frequently silenced via gene methylation in ESCC [16]. In keeping with this concept, treatment of two ESCC cell lines (*i.e.* EC109 and EC9706) with 5-aza-dC, a DNA methyltransferase inhibitor, restored the expression of E-cadherin (Figure 3B). Importantly, 5-aza-dC treatment also restored the regulatory control of E-cadherin expression by the miR-200b-ZEB1/2 axis (Figure 3C). In comparison, in an immortalized esophageal epithelial cell line NE2, in which the loss of E-cadherin has been shown to be unassociated with DNA hypermethylation [17], miR-200b mimic transfection could effectively induce E-cadherin expression (Figure 3A). Overall, these data suggests that an E-cadherin-independent mechanism may mediate the tumor suppressive effects of miR-200b in ESCC.

### miR-200b suppresses ESCC cell spreading and invasiveness via inhibiting the PI3K-AKT pathway

**Figure 4:**
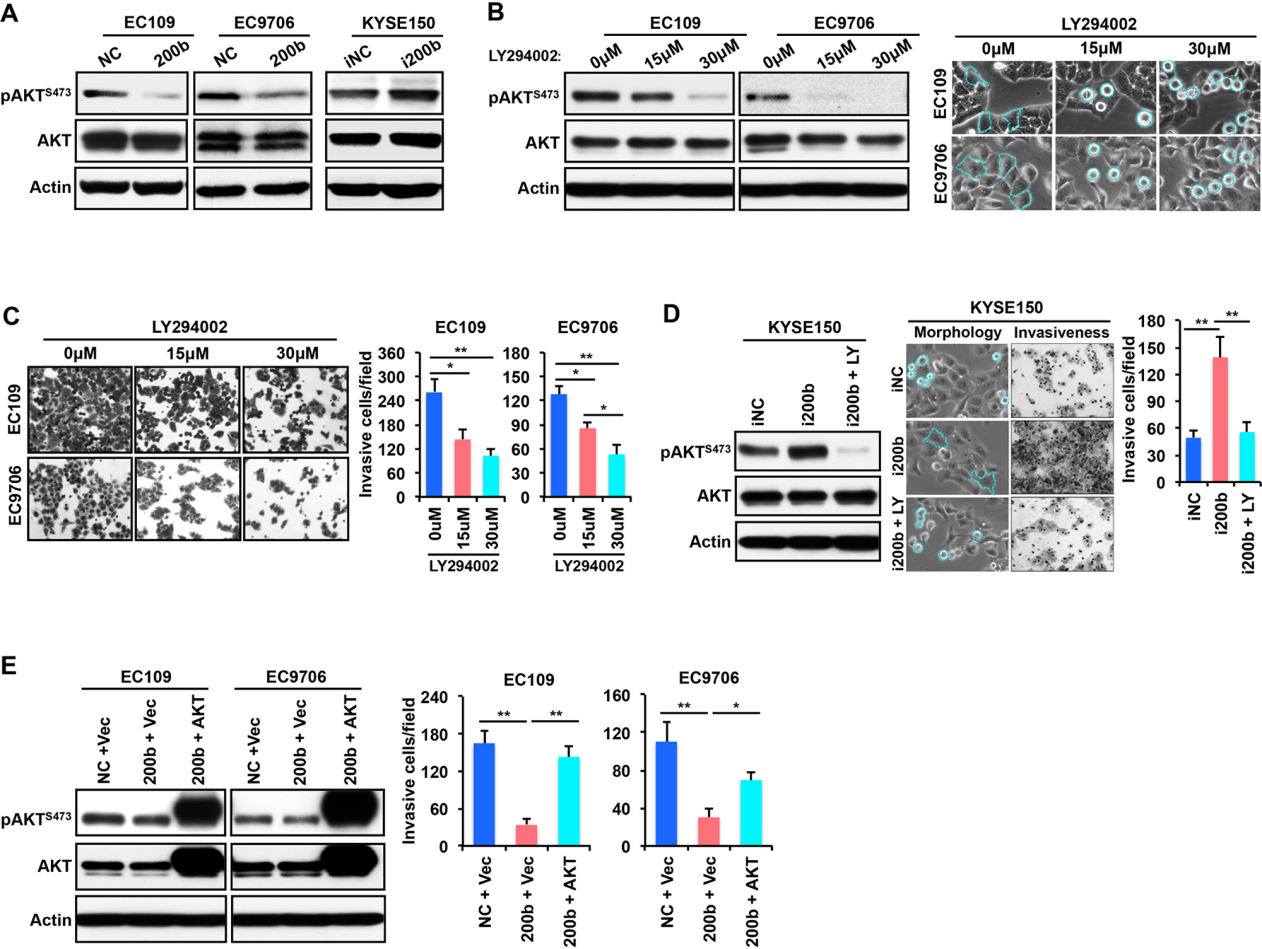
AKT mediates the biological effect of miR-200b in suppressing ESCC cell invasiveness **A.** The effects of miR-200b or miR-200b inhibitor (i200b) transfection on the expression of phospho-AKT^Ser473^ (pAKT^S473^) was determined by Western blot. Actin was used as a loading control. **B.** Left panel: the inhibitor effect of the PI3K inhibitor LY294002 on the expression of pAKT^S473^ was determined by Western blot. Actin was used as a loading control. Right panel: The impact of LY294002 on cell morphology. Dashed lines were used to note the morphological changes. **C.** The impact of LY294002 on cell invasiveness was detected using transwell invasiveness assays. Data are presented as mean ± SD. *P < 0.05, **P < 0.01, Student's *t* test. **D.** LY294002 (LY) suppresses the biological effects of miR-200b inhibitor (i200b) in KYSE150 cells. Data are presented as mean ± SD. **P < 0.01, Student's *t* test. **E.** A constitutively active form of AKT, myristoylated AKT (myr-AKT), restored cell invasiveness that was suppressed by miR-200b mimic transfection. Data are presented as mean ± SD. *P < 0.05, **P < 0.01, Student's *t* test.

Our previous study has shown in ESCC cells that miR-200b can induce a morphological change from a spindle-like shape to round cell morphology, and induce dramatic changes in cytoskeleton structures, *i.e*. a decrease in the formation of stress fibers and cell protrusions [11]. Since it has been reported that the PI3K pathway plays an important role in modulating actin cytoskeleton and cell morphology [18, 19], we assessed whether the phenotypic changes caused by miR-200b was linked to the PI3K pathway. As shown in Figure 4A, enforced expression of miR-200b decreased the expression of phospho-AKT^Ser473^ (pAKT), a key downstream mediator of the PI3K pathway, in both EC109 and EC9706 cells. In comparison, miR-200b inhibition in KYSE150, an ESCC cell line that expressed a relatively high level of miR-200b [11], substantially increased pAKT. Then, we assessed whether inhibition of the PI3K pathway using LY294002 can mimic the biological function of miR-200b in ESCC cells. As shown in Figure 4B–4C, LY294002 induced cell rounding and inhibited invasiveness in a dose-dependent manner in both EC109 and EC9706 cells. Moreover, the morphological changes and invasiveness enhanced by miR-200b inhibition in KYSE150 cells were dramatically retarded by LY294002 treatment (Figure 4D). Furthermore, as shown in Figure 4E, enforced expression of a constitutively active form of AKT, *i.e.* myristoylated AKT (myr-AKT), significantly restored invasiveness that was suppressed by miR-200b in both EC109 and EC9706 cells (*P* < 0.05).

### miR-200b suppresses the PI3K-AKT signaling via blocking the inside-out activation of integrin β1

**Figure 5:**
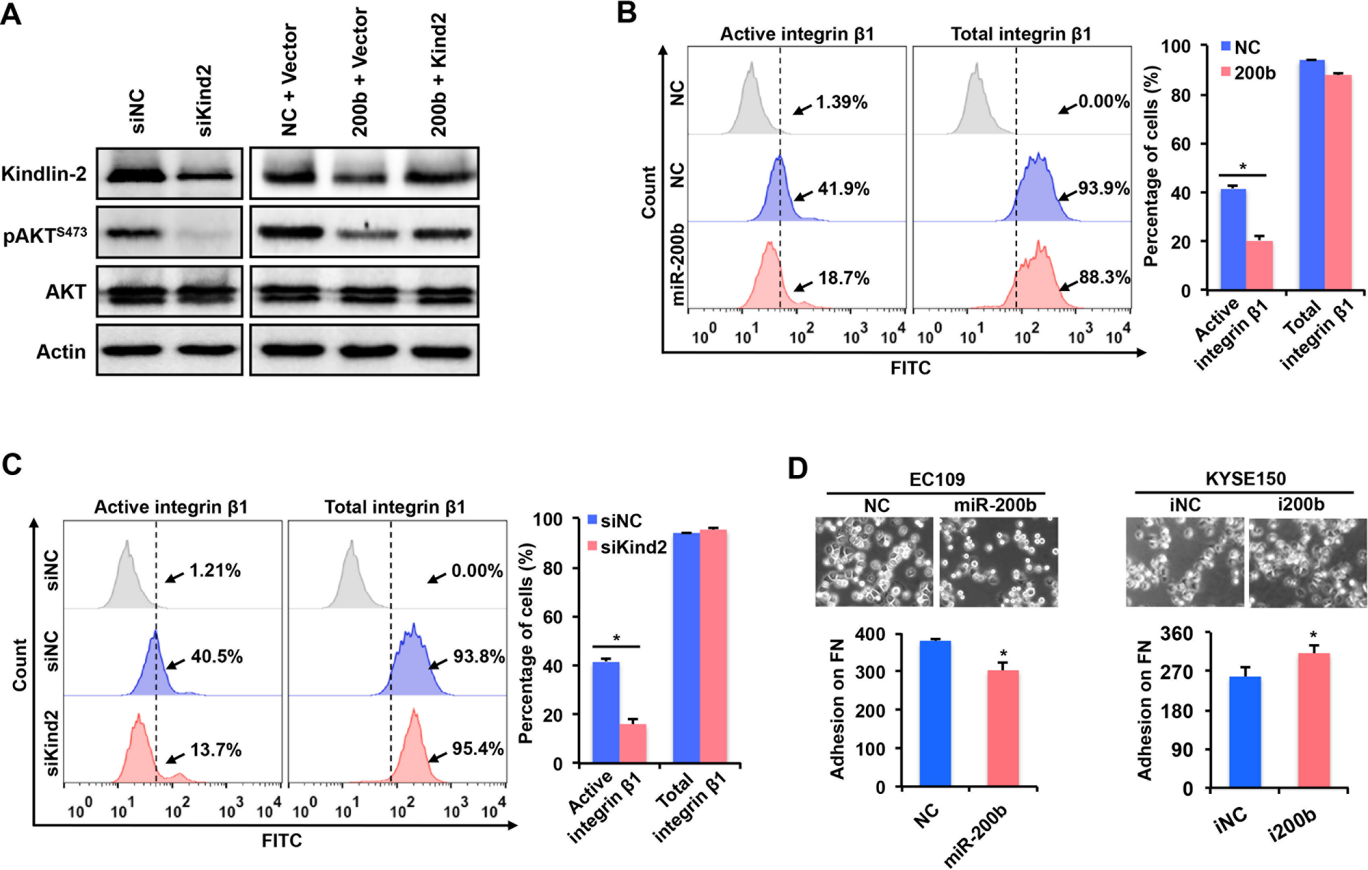
miR-200b inhibits the activation of AKT via suppressing the Kindin-2- integrin β1 axis **A.** In EC109 cells, Western blot was performed to determine the expression of Kindlin-2 and pAKT^S473^ after transfection. Actin was used as a loading control. **B–C.** In EC109 cells, flow cytometry was performed to assess the effects of enforced expression of miR-200b and Kindlin-2 knockdown on the expression of active/total integrin β1. Data are presented as mean ± SD. *P < 0.05, Student's *t* test. **D.** Cell adhesion assay was performed to determine the impact of enforced expression or inhibition of miR-200b on cell adhesion on fibronectin (FN). Data are presented as mean ± SD. *P < 0.05, Student's *t* test.

The mechanism underlying the suppression of the PI3K-AKT pathway by miR-200b remains unclear in ESCC. Since Kindlin-2, a target of miR-200b [11], is one of the key molecules mediating the inside-out activation of integrin β1 [20, 21], and integrin β1 has been shown to activate the PI3K-AKT pathway [18, 19, 22, 23], we hypothesized that the miR-200b can repress the Kindlin-2-integrin β1-AKT axis. Our data are in support of this hypothesis. First, as shown in Figure 5A, knockdown of Kindlin-2 dramatically decreased the expression of pAKT, and re-expression of Kindlin-2 restored the pAKT expression that was suppressed by miR-200b mimic transfection. Second, as shown in Figure 5B–5C, both enforced expression of miR-200b and Kindlin-2 knockdown significantly decreased the percentage of cells with active integrin β1, detected by flow cytometry. Third, as shown in Figure 5D, enforced expression of miR-200b significantly reduced, while inhibition of miR-200b significantly enhanced, cell adhesion on fibronectin (*P* < 0.05), a major ligand for integrin β1, indicating the inhibitory effect of miR-200b on the biological function of the Kindlin-2-integrin β1 axis. Taken together, these data suggest that miR-200b suppresses the PI3K-AKT pathway via inhibiting the Kindlin-2-integrin β1 axis.

### The Kindlin-2-integrin β1-AKT axis in ESCC patient samples

**Figure 6:**
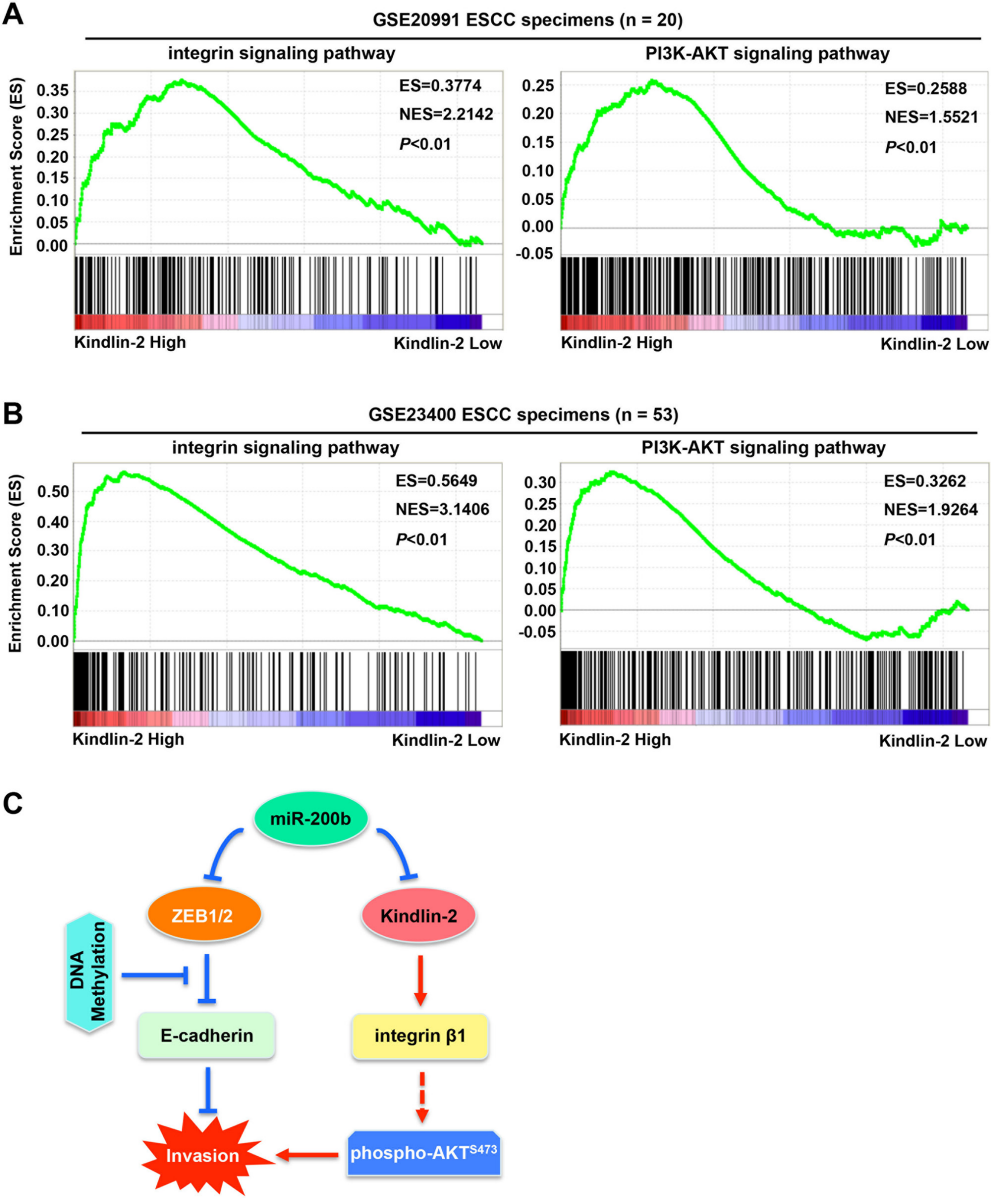
Correlation between Kindlin-2 and the integrin signaling pathway and the PI3K-AKT signaling pathway in ESCC tumors **A–B.** GSEA (Gene Set Enrichment Analysis) was performed to analyze the correlation between Kindlin-2 and the integrin signaling pathway or the PI3K-AKT signaling pathway in two independent cohorts of ESCC patients (NCBI/GEO/GSE20991, *n* = 20; and NCBI/GEO/GSE23400, *n* = 53). **C.** A schematic model showing the possible mechanisms underlying the invasiveness-suppressing role of miR-200b in ESCC. Specifically, *E-cadherin* gene methylation can block the control of E-cadherin expression by the miR-200-ZEB1/2 axis, indicating that an E-cadherin-independent mechanism can mediate the biological function of miR-200b. Our findings show that miR-200b can suppress the Kindlin-2-integrin β1-AKT pathway to mitigate ESCC cell invasiveness. Note that arrows indicate stimulation effects, while blunt arrows indicate inhibitory effects.

To validate whether the Kinlin-2-integrin β1-AKT axis is present in ESCC tumors, we performed GSEA (Gene Set Enrichment Analysis) [24, 25]. As shown in Figure 6A–6B, Kindlin-2 expression was positively correlated with the activation of both the integrin signaling pathway and the PI3K-AKT signaling pathway in two independent cohorts of ESCC patients (both *P* < 0.01, *n* = 20 and *n* = 53, respectively). Specifically, up-regulated target genes in both signaling pathways tend to be more enriched in tumors that expressed higher levels of Kindlin-2 in both cohorts of ESCC patients. Collectively, these data suggest that miR-200b suppresses the integrin β1-AKT pathway via targeting Kindlin-2, which may mediate the role of miR-200b in mitigating ESCC cell invasiveness.

## DISCUSSION

In a recent review paper [7], we have described that miR-200 plays a tumor suppressor role in multiple stages of tumor progression, including tumor initiation, invasion, metastasis and chemoresistance. To our knowledge, apart from our studies in ESCC [11, 26], only one study has reported the deregulation of miR-200 in the neoplastic diseases of the esophagus, which revealed a reduced expression of miR-200 in both Barrett's esophagus and esophageal adenocarcinoma [27]. However, the role of miR-200 in ESCC remains largely unclear. Since ESCC is a type of malignant cancer with a high frequency of local invasion and metastasis [3–6], we determined in this study the involvement of the miR-200-ZEB1/2 axis, one of the key EMT determinants [7, 13, 14], in the invasiveness of ESCC. We revealed a significant inverse correlation between the expression of miR-200b and ZEB1/2, suggesting the existence of this regulatory axis in ESCC, which may be attributed to the binding of miR-200b in the 3′UTR of ZEB1/2 [13, 14]. Deregulation of the miR-200-ZEB1/2 axis was found to contribute to the pathobiology of ESCC, as manifested by their prognostic values in ESCC patients. However, the suppression of invasiveness by miR-200b both *in vitro* and *in vivo* was unassociated with E-cadherin, a major downstream mediator of the miR-200b-ZEB1/2 axis, in certain ESCC cells. We revealed that epigenetic silencing of *E-cadherin* (*i.e*. DNA methylation) impaired the control of E-cadherin expression by the miR-200-ZEB1/2 axis, possibly by blocking the binding of ZEB1/2 to the promoter region of *E-cadherin* gene. This finding suggests that miR-200b can suppress ESCC cell invasion via E-cadherin or EMT independent pathways. In keeping with our hypothesis, we uncovered the Kindlin-2-integrin β1-AKT pathway as an important mediator of the biological function of miR-200b in ESCC cells (Figure 6C).

It has been reported that cancer cell invasion can be driven by cytoskeletal reorganization independent of EMT or E-cadherin [28–30]. The data presented in both the current study and our previous study [11] is in support of this concept. Specifically, miR-200b mitigated ESCC cell invasiveness and dramatically altered the cytoskeletal structure and morphology without affecting the expression of E-cadherin and vimentin. Recent findings from other studies are also in keeping with our observation [8–12]. For instance, miR-200 was shown to target cytoskeleton remodeling proteins in breast cancer cells, including WASF3, Moesin, FHOD1, PPM1F, WIPF1 and Cofilin2, functional mediators of miR-200 in the suppression of invasion and/or metastasis [8–12]. Notably, different from the case in ESCC, these studies showed that miR-200 impact both the classic ZEB1/2-EMT pathway and the cytoskeleton reorganization process in breast cancer cells [8–12].

Kindlin-2 has been shown to promote cancer cell migration and invasion, and multiple self-reinforcing feedback loops between Kindlin-2 and oncogenic tyrosine kinases have been reported. For instance, the reciprocal stimulation between the tyrosine kinase Src and Kindlin-2 has been shown to enhance cancer cell spreading and migration [31]. Similar with the Kindlin-2-integrin β1-AKT axis in ESCC cells, a Kindlin-2-EGFR-AKT axis has been revealed in breast cancer cells [32]. Interestingly, the EGFR-AKT signaling activated by Kindlin-2 can in turn augment Kindlin-2 expression, forming a self-reinforcing feedback loop that drives cell migration [32]. If the AKT signaling also activates Kindin-2 expression in ESCC cells, the Kindlin-2-integrin β1-AKT axis may also initiate a self-strengthening feedback loop to promote invasiveness, whereas further experimental evidence is needed to support this concept.

The PI3K/AKT signaling has been shown to be constitutively active in ESCC, which promotes ESCC tumor growth, invasion and chemoresistance [33–36]. The invasiveness-enhancing role of AKT was reported to be mediated by promoting TGF-β-induced EMT or stimulating the expression of MMP2 [35, 36]. Pharmacological blockade of the PI3K-AKT pathway or siRNA knockdown of AKT have been shown to mitigate ESCC cell invasiveness [35, 36]. In this study, we revealed miR-200b as an upstream suppressor of the PI3K-AKT pathway via blocking the Kindlin-2-integrin β1 axis. Thus, restoring miR-200b expression can be a promising approach to attenuate the PI3K-AKT pathway, hence, suppressing tumor aggressiveness in ESCC.

Integrin β1 is one of the most important integrin family members that plays important roles in promoting cytoskeleton remodeling, cell morphology, motility and invasiveness [18, 37, 38]. The activation of integrins is bidirectional, including the outside-in activation (activated by extracellular matrix components such as collagens and fibronectins) and the inside-out activation (activated by focal adhesion proteins such as Talins and Kindlins) [20, 21]. Our previous study has identified Kindlin-2, a significant prognostic marker in ESCC [39], as a target and mediator of the function of miR-200b in ESCC [11]. In this study, we revealed that miR-200b inhibits the inside-out activation of integrin β1 via targeting Kindlin-2. Downstream mediators of the biological function of integrin β1 include FAK and Rho GTPases, key regulators of cell morphology, adhesion and invasiveness [37, 38, 40, 41]. This is in keeping with our finding that miR-200b can significantly mitigate these functions in ESCC cells. Moreover, miR-200b has been shown to inhibit the activation of FAK and Rho GTPases via targeting Kindlin-2 [11]. Thus, this study suggests that integrin β1 is a protein that transmits signals downstream of the miR-200b-Kindlin-2 axis to regulate ESCC cell morphology, adhesion and invasiveness.

To conclude, our data suggest that deregulation of the miR-200-ZEB1/2 axis contributes to the pathobiology of ESCC, which can serve as prognostic markers in ESCC patients. DNA methylation of the *E-cadherin* gene may block the control of E-cadherin expression by the miR-200b-ZEB1/2 axis in ESCC, whereas miR-200b can suppress invasiveness via inhibiting the Kindlin-2-integrin β1-AKT signaling cascade.

## MATERIALS AND METHODS

### Patients and clinical samples

Human ESCC samples and adjacent non-tumor tissues were collected directly after surgical resection at the Department of Tumor Surgery of Shantou Central Hospital, China. The samples were immediately frozen in liquid nitrogen and stored at −70°C or fixed in 10% formalin for paraffin embedding. The cases were selected based on a clear pathological diagnosis, follow-up data, and had not received previous local or systemic treatment. Patients were staged in accordance with the 7th edition American Joint Committee on Cancer tumor-nodes-metastasis (AJCC TNM) staging system. The study was approved by the ethical committee of Shantou Central Hospital and the ethical committee of the Shantou University Medical College. Written informed consent was obtained from all surgical patients to use resected samples for research.

### Cell culture

Details of the human ESCC cell lines used in this study have been described previously [11]. Additional human ESCC cell lines KYSE140 and COLO680N were cultured in RPMI-1640 medium supplemented with 10% fetal bovine serum, while SHEE, EC171, EC18, and EC8712 were cultured in 199 medium supplemented with 10% newborn calf serum.

### *In vivo* tumor invasion assay

This animal study was conducted using the protocol described in our previous study [15]. Specifically, EC109 cells transfected with 20 nM miR-200b mimic or negative control RNA (denoted as EC109-NC and EC109–200b) were trypsinized and suspended in PBS. 50 μl cell suspensions containing 1 × 10^6^ cells were injected into the left footpads of 5-week-old male BALB/c nu/nu mice (*n* = 10 per group). After 23 days, the mice were sacrificed. The invasive length was measured from the injection site in the footpad to the furthest visible invasion site. The width and height of the invasive area were also measured using a vernier caliper. Tumor local invasion area was measured by multiplying the length, width, and height. The primary tumors in the footpad and the invasive tumors in the inner thigh muscles were excised and embedded in paraffin after fixation in 10% formaldehyde/PBS. Subsequently, the tissue sections were stained with haematoxylin-eosin and examined for the existence of invasive carcinoma. E-cadherin and vimentin expression was analyzed in the primary tumors by immunohistochemistry. The animal experiments were performed in accordance with the Institutional Animal Care and Use Committee of Shantou University.

### Chemical treatment

Both the DNA methyltransferase inhibitor 5-aza-2′-deoxycytidine (5-aza-dC) and PI3K inhibitor LY294002 were purchased from Sigma-Aldrich. 5-aza-dC was dissolved in acidic acid, while LY294002 was dissolved in DMSO. For 5-aza-dC treatment, cells were cultured in medium containing different concentrations of this chemical for 72 h before RNA extraction or cell lysate collection. The culture medium was changed every 24 h with corresponding concentrations of 5-aza-dC. For LY294002 treatment, cells were incubated with medium containing different concentrations of this chemical for 24 h before cell lysate harvest or biological function study.

### RNA isolation and real-time PCR

Total RNA was extracted using TRIzol reagent (Invitrogen, Carlsbad, CA). For the real-time PCR of miR-200a, miR-200b, miR-429 and RNU6B (U6, endogenous control), TaqMan MicroRNA Assay kits (Applied Biosystems, Foster City, CA) were used and real-time PCR reaction was carried out using ABI 7500 fast real-time PCR system (Applied Biosystems) as described before [11]. For the detection of ZEB1, ZEB2 (using real-time PCR), E-cadherin, vimentin and GAPDH (using RT-PCR), cDNA was synthesized with the Reverse Transcription System (Promega, Madison, WI). The sequences of the primers are described in Supplementary Table 1.

### Plasmid, miRNA, and siRNA and transfection

The construction of the pcDNA3-Kindlin-2 plasmid was described in our previous study [11]. The pcDNA3-Myr-HA-Akt1 plasmid was a gift from William Sellers (Addgene plasmid #9008). The miR-200b mimic (hsa-miR-200b-3p), miR-200b inhibitor, and Kindlin-2 siRNAs were purchased from Qiagen as previously described [11]. The transfection of plasmids, miR-200b mimic, miR-200b inhibitor, and Kindlin-2 siRNAs has been described previously [11].

### Western blot

Proteins were separated on SDS-PAGE and transferred to PVDF membrane (Millipore, Bedford, MA). The membranes were blocked with 5% non-fat milk and incubated with antibodies against E-cadherin (1:400, Santa Cruz Biotechnology, Santa Cruz, CA), vimentin (1:500, Dako, Carpinteria, CA), Kindlin-2 (1:2000, Millipore), phospho-AKT^S473^ (1:1000, Cell Signaling), AKT (1:1000, Cell Signaling), and β-actin (1:10000, Sigma, St. Louis, MO, USA). The proteins were detected with Western Blot Luminol Reagent (Santa Cruz Biotechnology).

### Immunohistochemistry (IHC)

Paraffin embedded tissue blocks were cut into 4 μm sections and processed for IHC with a protocol described previously [42]. Anti-E-cadherin (1:1, Zhongshan Golden Bridge Biotechnology, Beijing, China) was used in IHC studies. Scoring was classified into 4 grades: no reactivity scored 0, faint reactivity scored 1, moderate reactivity scored 2, and strong reactivity scored 3.

### Invasion and adhesion assays

Cell invasion assays were performed as described in our previous studies [11, 15]. For adhesion assays, BioCoat Cellware (96-well) pre-coated with human fibronectin (Corning) was used. After washing the wells three times with PBS, the wells were blocked for 1 h at 37°C with 1% BSA (bovine serum albumin) dissolved in water. Wash the wells twice with PBS, and 3 × 10^4^ cells diluted in 100 μl media were plated in each well. After incubating the plates at 37°C for 15 min, unattached cells were removed by gently washing the cells twice with warm media. Add 100 μl media to each well, and take photos of the adherent cells under microscope.

### Flow cytometry

The expression of active and total integrin β1 was determined by flow cytometry. Briefly, cells were gently dissociated with 0.05% trypsin and 0.02% EDTA, washed in medium containing 1% BSA, counted and diluted in PBS. 5 μl antibody against active integrin β1 (HUTS-4-FITC, Millipore) and 2.5 μl antibody against total integrin β1 (Santa Cruz Biotechnology) was added to 95 μl PBS containing 5 × 10^5^ cells for the detection of active integrin β1 and total integrin β1, respectively. These cells were incubated for 30 min at room temperature. No antibody was added in the negative control cells. The cells incubated with antibody against total integrin β1 were then incubated with FITC-conjugated secondary antibody (goat-anti-mouse, Santa Cruz Biotechnology) for 30 min on ice. Then, all the samples were washed in cold PBS and analyzed by flow cytometry. Data were analyzed using the Flow Jo software.

### Statistical analysis

All statistical analyses were performed using the SPSS V.13.0 statistical software package or the Graphpad Prism version 6. Student's *t* test was used to compare the difference between two independent groups of values. Survival curves were plotted using the Kaplan-Meier method and compared using the log-rank test. Differences were considered significant when the *P* value was less than 0.05.

## SUPPLEMENTARY TABLE AND FIGURES


