# Supplementary material for: Loss of miR-200b promotes invasion via activating the Kindlin-2/integrin β1/AKT pathway in esophageal squamous cell carcinoma: An E-cadherin-independent mechanism
Source: Oncotarget. 2015 Aug 20;6(30):28949–60. doi: 10.18632/oncotarget.5027 (PMC4745703; doi:10.18632/oncotarget.5027)
Supplement: Supplementary file 1 [file oncotarget-06-28949-s001.pdf]

SUPPLEMENTARY TABLE AND FIGURES

Supplementary Table S1: Primers for RT-PCR and real-time PCR

Primer Name	Application	Primer Sequence
ZEB1-F	real-time PCR	TTCAAACCCATAGTGGTTGCT
ZEB1-R	real-time PCR	TGGGAGACACCAAACCAACTG
ZEB2-F	real-time PCR	CAAGAGGCGCAAACAAGC
ZEB2-R	real-time PCR	GGTTGGCAATACCGTCATCC
E-cadherin-F	RT-PCR	CACACGGGGCGAGTGCCAAC
E-cadherin-R	RT-PCR	GCGGCCCCTTCACAGTCACA
vimentin-F	RT-PCR	GATGCCCTTAAAGGAACCAATGAG
vimentin-R	RT-PCR	GGCGGCCAATAGTGTCTTGGTAG
GAPDH-F	RT-PCR	GGCGGCCAATAGTGTCTTGGTAG
GAPDH-R	RT-PCR	GAAGATGGTGATGGGATTTC

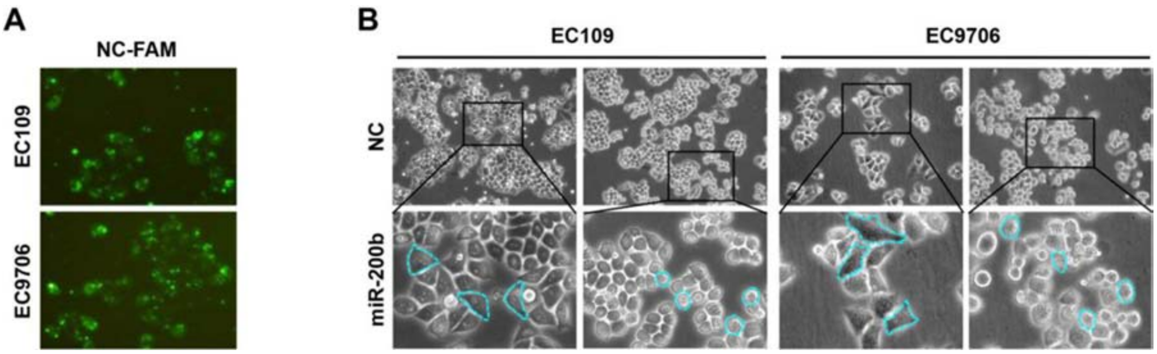

Supplementary Figure S1: A. To evaluate the transfection efficiency, negative control miRNA labeled with fluorescein amidite (NC-FAM) was transfected into EC109 and EC9706 cells. The strong green fluorescence inside the cells indicate a high transfection efficiency using our protocol. **B.** EC109 and EC9706 cells were transfected with either a miR-200b mimic or NC, and the influence of miR-200b transfection on cell morphology was shown. Dashed lines were used to highlight the morphological changes.

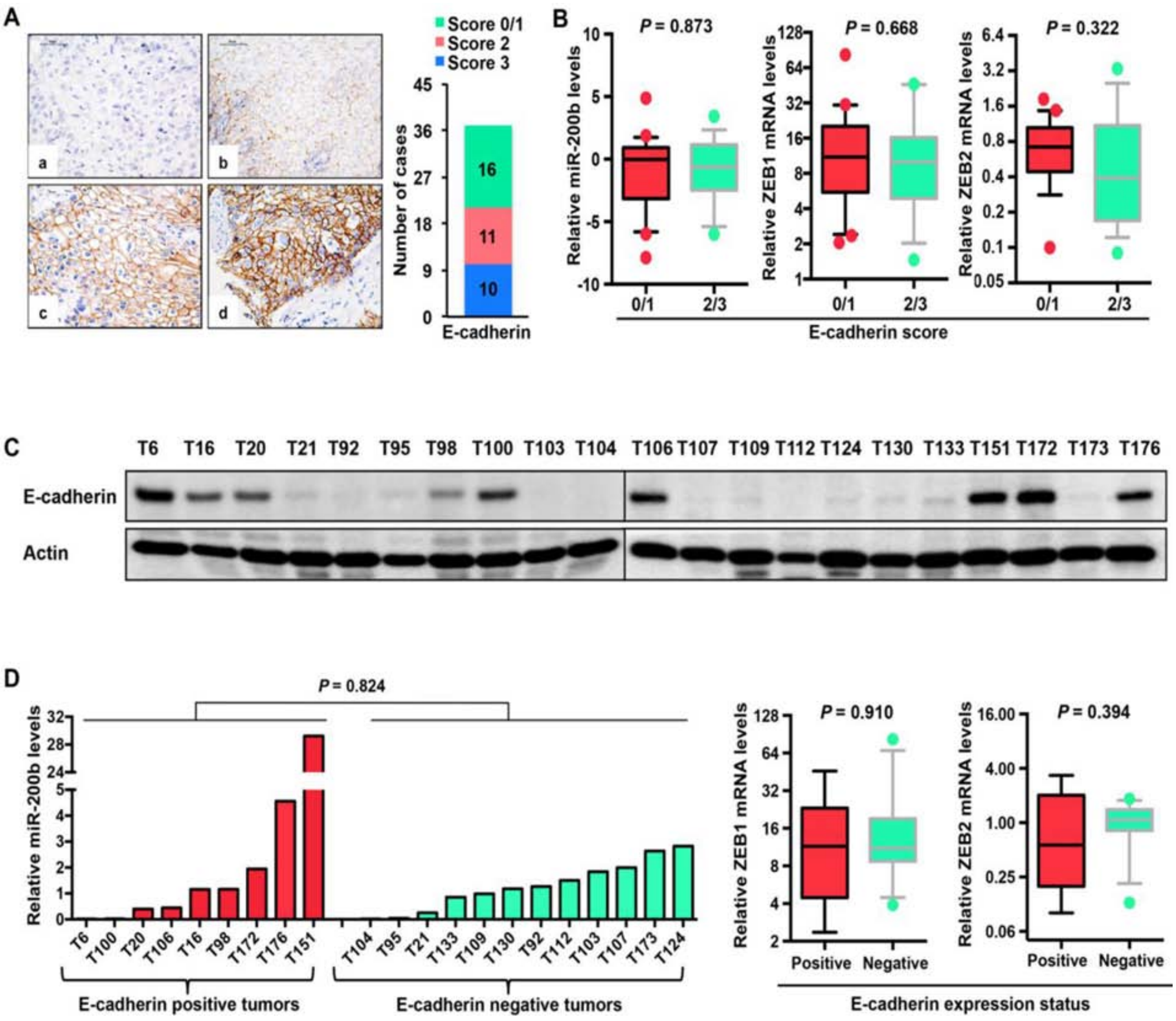

Supplementary Figure S2: The expression levels of E-cadherin do not correlate with miR-200b or ZEB1/2 in ESCC tumors. A. The expression of E-cadherin was detected using immunohistochemistry in 37 cases of ESCC specimens. Left panel: representative images show: (a) negative staining, score 0; (b) weak staining, score 1; (c) moderate staining, score 2; (d) strong staining, score 3. Right panel: the proportion of tumors displaying different E-cadherin staining intensities. **B.** The expression of miR-200b and ZEB1/2 were compared between ESCC tumors with different staining intensities of E-cadherin. Box plots are presented as 10–90 percentile. Statistical analysis was performed using Student's *t* test and Mann Whitney *U* test for miR-200b and ZEB1/2, respectively. **C.** Western blot was performed to determine E-cadherin expression in 22 cases of frozen tumors randomly chosen from the 37 cases of ESCC tumors described in (A) Actin was used as a loading control. **D.** The expression of miR-200b and ZEB1/2 were compared between ESCC tumors with different expression levels of E-cadherin as determined by Western blot shown in (C) Statistical analysis was performed using Student's *t* test and Mann-Whitney *U* test for miR-200b and ZEB1/2, respectively. Box plots are presented as 10–90 percentile.
